# Large-Scale Genotyping-by-Sequencing Indicates High Levels of Gene Flow in the Deep-Sea Octocoral *Swiftia simplex* (Nutting 1909) on the West Coast of the United States

**DOI:** 10.1371/journal.pone.0165279

**Published:** 2016-10-31

**Authors:** Meredith V. Everett, Linda K. Park, Ewann A. Berntson, Anna E. Elz, Curt E. Whitmire, Aimee A. Keller, M. Elizabeth Clarke

**Affiliations:** 1 National Research Council, under contract to Northwest Fisheries Science Center, National Marine Fisheries Service, NOAA, Seattle, Washington, United States of America; 2 Conservation Biology Division, Northwest Fisheries Science Center, National Marine Fisheries Service, NOAA, Seattle, Washington, United States of America; 3 Fishery Resource Analysis and Monitoring Division, Northwest Fisheries Science Center, National Marine Fisheries Service, NOAA, Seattle, Washington, United States of America; 4 Office of the Science Director, Northwest Fisheries Science Center, National Marine Fisheries Service, NOAA, Seattle, Washington, United States of America; King Abdullah University of Science and Technology, SAUDI ARABIA

## Abstract

Deep-sea corals are a critical component of habitat in the deep-sea, existing as regional hotspots for biodiversity, and are associated with increased assemblages of fish, including commercially important species. Because sampling these species is so difficult, little is known about the connectivity and life history of deep-sea octocoral populations. This study evaluates the genetic connectivity among 23 individuals of the deep-sea octocoral *Swiftia simplex* collected from Eastern Pacific waters along the west coast of the United States. We utilized high-throughput restriction-site associated DNA (RAD)-tag sequencing to develop the first molecular genetic resource for the deep-sea octocoral, *Swiftia simplex*. Using this technique we discovered thousands of putative genome-wide SNPs in this species, and after quality control, successfully genotyped 1,145 SNPs across individuals sampled from California to Washington. These SNPs were used to assess putative population structure across the region. A STRUCTURE analysis as well as a principal coordinates analysis both failed to detect any population differentiation across all geographic areas in these collections. Additionally, after assigning individuals to putative population groups geographically, no significant F_ST_ values could be detected (F_ST_ for the full data set 0.0056), and no significant isolation by distance could be detected (p = 0.999). Taken together, these results indicate a high degree of connectivity and potential panmixia in *S*. *simplex* along this portion of the continental shelf.

## Introduction

Deep-sea coral communities are increasingly acknowledged as critical components of deep-sea ecosystems. They are recognized as regional hotspots for biodiversity, and appear to be associated with increased assemblages of fish, including commercially important species [1]. These communities are difficult to access, however, so a thorough understanding of some of the most fundamental biological and ecological features such as taxonomic relationships among corals and even basic species identification is lacking. Despite their relative inaccessibility to science, deep-sea corals are continually subjected to negative impacts from human activities such as trawl and pot fisheries, oil and gas exploration, sand and mineral mining, coral harvest, and cable and pipeline deployment, as well as ecological pressures such as climate change, ocean acidification, and invasive species [2, 3]. Information regarding basic life history and ecology is critical to assess the effects of such disturbances on these deep-sea coral communities, and in many regions of the world’s oceans even preliminary species inventories are lacking. Despite recent efforts in the mapping and identification of deep-sea coral communities [4–8], much remains unknown, including their degree of genetic connectivity. A compiled survey of coral species found in the Gulf of Alaska, Bering Sea, and Aleutian Islands listed 141 unique species, 52 of which lacked complete taxonomic identifications [9]. A similar survey from the Pacific Coast listed 101 unique taxa, 24 of which were not identified to species[2]. Undoubtedly some of these are species new to science.

Life history variation can have a large effect on the distribution of corals, and their ability to recover from both natural and anthropogenic disturbances [10–13]. Corals exhibit a diversity of life history traits including reproductive modes (see [14] for a review of reproduction in octocorals), and population structures. In some coral species, patterns of spatial population structure follow expectations of life history traits: individuals that are broadcast spawners disperse further, resulting in higher gene flow between populations, which reduces population differentiation [11, 13]. In contrast, species that brood their larvae can have more limited recruitment distances, resulting in lower levels of gene flow and subsequently higher levels of genetic structure [11, 13, 15]. However, there are exceptions to this pattern and dispersal of larvae can be influenced by both biological and physical factors including larval behavior, larval duration, mortality, reproductive timing, oceanographic circulation patterns, and availability of suitable settlement habitat [11, 16, 17].

The plexaurid gorgonian, *Swiftia simplex*, is found in the deep-sea (below 100 m [18]) across the western coast of North America [18–20]. *S*. *simplex* was originally identified in 1909 (Nutting), and initially assigned to the genus *Psammogorgia* based on colony morphology. While this nomenclature is still found in some literature and databases (for example, the World Register of Marine Species (WORMS) [21]), the sclerites of this taxa do not match those found in *Psammogorgia* as currently defined, and recent publications place the group in the genus *Swiftia* [18, 22]. *S*. *simplex* is gonochoric, and recent evidence suggests it is a broadcast spawner, as no brooding or larvae have been observed in any of the samples examined [23]. Additionally, Feehan and Waller [23] found *S*. *simplex* females had the highest fecundity, calculated as the count of previtellogenic and vitellogenic oocytes within the imaged polyps, of any of the eight octocoral species analyzed.

Given the complexities involved in tracking planktonic larvae, indirect methods for measuring connectivity, including both hydrodynamic modeling and genetics, currently provide the best predictions of dispersal and connectivity [24]. To date, much of the genetic work carried out on deep-sea corals has been devoted to distinguishing possible taxonomic divisions [5–8]. Studies examining genetic diversity, population structure, and connectivity within species have focused mainly on mitochondrial sequence data or small panels of microsatellites [16, 17, 25–27]. The complexity of obtaining samples from these remote and difficult to access coral communities remains a barrier to the extensive sampling generally needed for studies with these types of markers [5, 17, 26]. Thus the development of population-level markers, particularly large panels of markers capable of overcoming the need for large numbers of samples, is a necessary step toward a comprehensive understanding of the ecology and connectivity of these organisms.

The advent of high-throughput genotyping-by-sequencing techniques, including restriction-site associated DNA (RAD)-tag sequencing, has greatly expanded the speed and availability of genome-wide marker development in non-model organisms [28]. RAD-tag sequencing has been effectively used in studies of phylogeography and population genomics [29, 30] to show low but significant variation amongst groups indistinguishable with other marker types [31–33], reveal local selection [34], and detect cryptic species [7, 8]. The goals of this study were twofold: to develop novel molecular resources for the study of the ecology of *Swiftia simplex* through RAD-tag sequencing, and to apply the SNPs developed in this process to define the population structure and connectivity of this species across a portion of the United States West Coast.

## Materials and Methods

### Sample Collection and DNA Extraction

**Fig 1:**
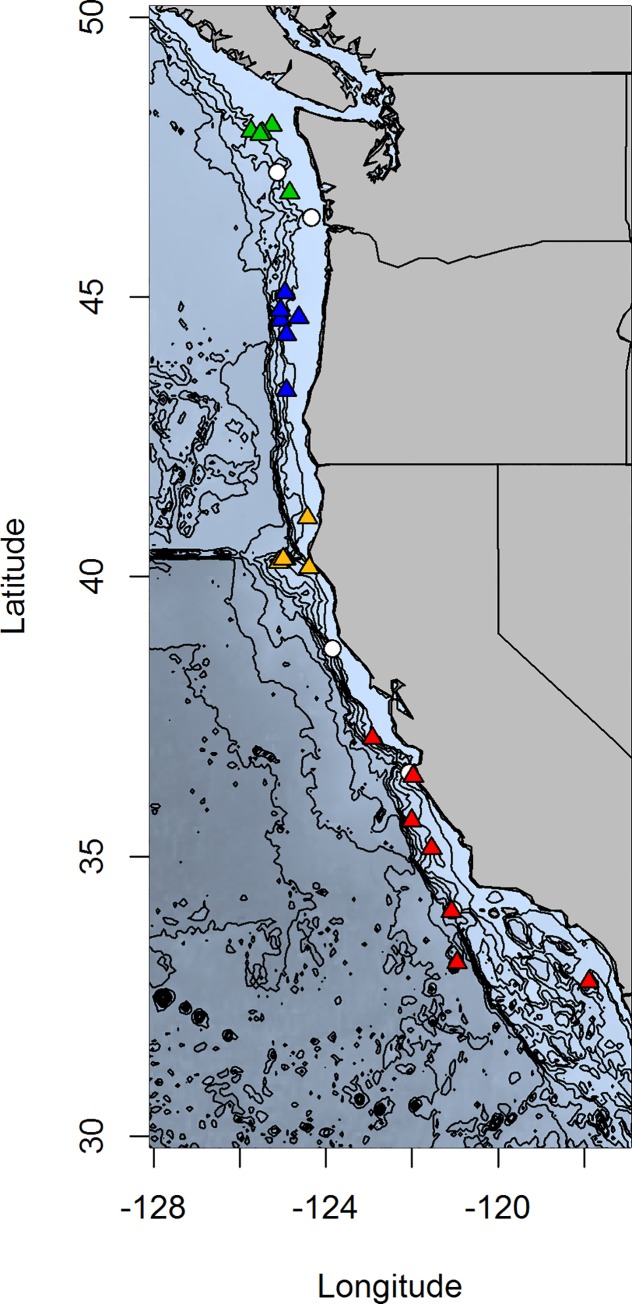
Map of collection locations for all 29 individuals initially included in this study. Colored triangles indicate individuals included in the population analysis, with the individual colors representing putative geographic populations. Population one individuals are red, two are gold, three are blue and four are green. White circles are individuals that failed to pass quality control and were excluded from the population analysis.

The *S*. *simplex* individuals included in this study came from three sources: the NOAA West Coast Upper Continental Slope Groundfish Trawl Survey (RACE), the NOAA West Coast Groundfish Bottom Trawl Survey (WCGBTS), and seamount surveys carried out by the Monterey Bay Aquarium Research Institute (MBARI). The specimens provided by the trawl surveys were collected as a regular part of NOAA’s annual WGCBTS program (RACE was a previous iteration of this survey). The permits for these collections were issued by the California Department of Fish and Game, the National Marine Sanctuaries, NOAA, Oregon State, and U.S. Fish and Wildlife. Detailed descriptions of the two trawl survey methods and goals can be found in Keller et al. [35] and Lauth [36]. Individuals obtained during the course of the trawl surveys were photographed and preliminarily identified by a shipboard biologist; a fragment of each individual was also preserved in 95% ethanol for later genetic analysis. The specimens provided by MBARI came from the archival collection maintained by that institution. Methods used to collect and preserve individuals via remotely operated vehicle (ROV) during the MBARI seamount surveys can be found in Lundsten et al. [20]. A total of twenty-nine (*n = 29*) individuals spanning the United States West Coast (Fig 1) were initially included in this study. The sample location, depth, and collecting agency for each individual can be found in Table 1. Trawl survey specimens are housed in ethanol storage or frozen in our laboratory (Table 1 has specimen numbers), while the original MBARI samples from which we obtained fragments are maintained in their collection.

**Table 1:** Collection metadata for the twenty-nine individuals initially included in this study.

Individual	Putative Population	Collecting Institution	Collection Date	Latitude	Longitude	Collection Depth (m)	NCBI Accession Numbers
**100203797**	1	WCGBTS	7/15/2011	32.76	-117.89	504.8	KX905019
**T665-A5**	1	MBARI	5/2/2004	33.11	-120.96	792.8	KX904983, KX905006
**T630-A13**	1	MBARI	10/16/2003	34.02	-121.08	895.3	KX904982, KX905005
**T631-X9**	NA	MBARI	10/16/2003	34.05	-121.04	722.6	KX904981, KX905004
**100186501**	1	WCGBTS	10/5/2008	35.14	-121.54	636.8	KX904987, KX905013
**100186506**	1	WCGBTS	10/3/2008	35.65	-122.01	1206.3	KX904988, KX905014
**100120887**	1	WCGBTS	10/3/2008	36.44	-121.97	120.8	KX905012
**100124450**	NA	WCGBTS	6/30/2008	36.5	-122.06	886.8	KX905017
**100158048**	1	WCGBTS	6/29/2007	37.12	-122.92	642.4	KX905011
**100101329**	NA	WCGBTS	2006	38.71	-123.84	486.7	KX904984, KX905007
**100231811**	2	WCGBTS	9/17/2012	40.16	-124.39	321.7	KX904998
**100230476**	2	WCGBTS	9/16/2012	40.26	-125.1	1191.4	KX904996
**100166577**	NA	WCGBTS	6/22/2010	40.31	-124.97	1040.6	KX904992
**100112079**	2	WCGBTS	6/21/2007	40.32	-124.99	1062.8	KX904986, KX905009
**100231801**	2	WCGBTS	9/15/2012	40.32	-124.94	904.9	KX904997
**100179316**	2	WCGBTS	9/22/2009	41.06	-124.43	469.3	KX904979, KX905003
**100241727**	3	WCGBTS	9/7/2012	43.33	-124.91	648.5	KX905001
**100241723**	3	WCGBTS	9/3/2012	44.33	-124.92	589.7	KX905000
**100203746**	3	WCGBTS	6/5/2011	44.59	-125.05	1194.3	KX904993
**100203749**	3	WCGBTS	6/5/2011	44.64	-124.63	252.5	KX904994
**21, 1996–15, 130**	3	RACE	11/3/1996	44.77	-125.06	1159.0	KX904980, KX905018
**100222555**	3	WCGBTS	8/21/2010	45.08	-124.94	867.3	KX904995
**100158022**	NA	WCGBTS	5/26/2007	46.42	-124.34	74.7	KX905010
**100165714**	4	WCGBTS	5/25/2010	46.86	-124.84	161.8	KX904991
**100105535**	NA	WCGBTS	8/20/2007	47.23	-125.13	837.7	KX904985, KX905008
**100186554**	4	WCGBTS	8/25/2008	47.91	-125.52	462.9	KX904990,KX905016
**100186551**	4	WCGBTS	8/25/2008	47.93	-125.47	357.7	KX904989, KX905015
**100179291**	4	WCGBTS	8/30/2009	47.97	-125.74	813.3	KX904978, KX905002
**100232856**	4	WCGBTS	5/25/2012	48.07	-125.25	147.2	KX904999

Samples obtained came from three primary sources: the NOAA West Coast Groundfish Bottom Trawl Survey (WCGBTS) [35], the NOAA West Coast Upper Continental Slope Groundfish Trawl Survey (RACE) [36], the 2003 and 2004 Seamount Expeditions carried out by the Monterey Bay Aquarium Research Institute (MBARI) [20]. Population groupings can be viewed on the map in Fig 1. Samples with NA designations were not included in the population analysis (see methods).

DNA was extracted from each sample using a Qiagen DNeasy 96 Kit on a Qiagen BioRobot 8000. Manufacturer’s protocols were followed throughout the DNA extraction and sequencing process unless otherwise noted. DNA samples were visualized on a gel to check overall DNA quality, and to match DNA qualities in downstream pools (see below).

### Species Confirmation and Phylogenetic Analysis

To confirm that all individuals were *Swiftia simplex* and to examine regional variation in mitochondrial haplotype, the mitochondrial sequences for the regions *MutS* and *igr+COI* were Sanger sequenced [6]. Analyses were performed on each mitochondrial sequence independently, as well as on their concatenation (*MutS+igr+COI*) whenever possible. Amplicons that were successfully sequenced were compared to a collection of sequences from voucher specimens identified by morphological taxonomists using phylogenetic analyses to confirm species identification (S1 Table). Sequence analysis was carried out using MEGA v. 5.2 [37]. First, sequences were aligned using ClustalW, and the alignments were checked and corrected by eye. Next, phylogenetic trees were constructed to confirm species identification using both maximum parsimony and maximum likelihood methods. The model test module in MEGA selected the Tamura 3-parameter model plus the gamma distribution (T92+G) for the maximum likelihood analysis. Each phylogenetic analysis was run with a bootstrap resampling parameter of 1,000.

### RAD-tag Sequencing and SNP Discovery

After species confirmation, total DNA concentrations were quantified using an Invitrogen Quant-iT PicoGreen dsDNA assay and an FLX800 fluorometer. DNA concentrations were initially low, so DNA from all individuals was concentrated on a Millipore MultiScreen_HTS_ PCR 96-well plate, after which a second quantification was carried out as previously noted.

After DNA quantification, three individuals were excluded due to low DNA concentration (<500ng total DNA yield). RAD-tag library preparation was subsequently carried out on the remaining twenty-six individuals, following the methods described in Baird et al. [38]. Briefly, DNA from each individual was digested using *SbfI*. Illumina sequencing adapters and 6 bp barcodes (a unique barcode for each individual) were ligated to each individual (barcodes for each individual are included in S2 Table). Barcoded samples were then pooled, matching approximate DNA qualities to minimize potential bias. Pooled samples were sheared, and each library amplified via PCR. Library quality and concentration were assayed via qPCR with a KAPA Biosystems Library quantification kit. The RAD-tag library was sequenced on an Illumina HiSeq 2000 at the University of Oregon High Throughput Sequencing Facility in a single end sequencing run using the 100 bp configuration. Initial data quality of the RAD-tag libraries was checked using FastQC v. 11.03 [39]. The Stacks software package (v. 1.23) [40, 41] was used to discover and genotype SNPs. First, sequences were quality filtered, demultiplexed, and trimmed using the *process_radtags*. Sequences were trimmed to 95bp based on the FastQC output. After running *process_radtags*, three individuals with extremely low sequence coverage (just 409 to 16,194 total reads per individual) were identified and removed from further analysis. The remaining twenty-three individuals were run through the remaining Stacks pipeline: *ustacks*, *cstacks*, *sstacks* and *populations*. To choose optimal parameters, *ustacks* and *cstacks* were run multiple times, varying the values of *m*, the minimum number of reads to create a stack, from 2 to 6, and *n*, the number of mismatches allowed between stacks when creating the catalog, from 0 to 3. Results of these runs were compared and final parameters were selected. In all *ustacks* runs the–*r* and–*D* options were used to limit highly repetitive stacks. Ultimately, *ustacks* was run with *m =* 5 and a catalog was constructed from this output in *cstacks* with *n =* 2. As a limited number of individuals were included in this study, all individuals were included in the catalog. Next, *ustacks* was run again, with *m =* 3, in order to improve genotyping accuracy in low coverage individuals. This output was compared to the catalog using *sstacks*. Finally, genotypes were called using *populations* with *m*, the minimum stack depth to include a locus equal to 7, and grouped individuals into four putative populations based on sample location (Fig 1, Table 1). Genotypes were output in VCF format for downstream filtering and analysis.

All filtering of SNP loci was carried out using vcftools v. 1.12b. PGDSpider (v. 2.0.5.2) [42] was used to convert VCF files to all other formats, including GENEPOP [43], STRUCTURE [44, 45] and Arlequin [46]. First, RAD-tags containing more than three SNPs per tag as well as singletons, SNPs occurring in only a single individual, were identified and removed from further analysis. Three individuals had noticeably more missing markers than the others (average number missing was 2,394 in these individuals versus 1,526 for the others, S2 Table). Examination of the raw data files showed that these two out of the three individuals had low initial sequence coverage overall, possibly due to sample quality. To test for any effects of the trade-off between utilizing fewer markers in all twenty-three individuals versus using a larger set of SNPs in fewer individuals, two data subsets were created at this stage: one with all twenty-three individuals and one where the three individuals with the most missing markers removed (n = 20). The following filtering steps were then carried out on each data set. The data were filtered to remove loci with minor allele frequencies <0.05 across all individuals. Tests for Hardy-Weinberg equilibrium were carried out in GENEPOP (v. 4.3), and loci out of equilibrium (P < 0.05) in two or more putative populations were removed. Next, the data were filtered to remove any SNP loci that failed to genotype in at least 80 percent of individuals. Finally, to reduce linkage in the data set for STRUCTURE and other analyses for which linkage violates the underlying assumption of the model, a subset of the final SNPs was generated containing only the first SNP in any tag containing more than one.

### Population Analysis

Initial analysis of putative population structure was carried out in STRUCTURE (v. 2.3.4). K values were varied from 1–6, using a burn in of 500,000 replicates followed by an additional 500,000 MCMC repeats. Each K value was tested 10 times. An admixture model was selected, using the putative population groupings based on region as a prior. STRUCTURE output was examined to determine optimal K values using Structure Harvester [47], and where appropriate STRUCTURE plots were generated with CLUMPP and Distruct to look for patterns. To test the potential effect of missing loci on the analysis, additional STRUCTURE runs were conducted on loci that genotyped in >90% and >95% of individuals using the same parameters. Additionally, a principal components analysis (PCA) was carried out using the R package adegenet [48]. Pairwise F_ST_ values and significance tests between all putative populations were carried out in Arlequin (v. 3.5) with 10,000 permutations and a significance level of p<0.05. An AMOVA was also carried out in Arlequin to examine the variance within and between populations, and significance was calculated via a 10,000 permutation test, p<0.05. GenAlEx (v. 6.205) [49] was used to calculate the observed vs. expected heterozygosities for all loci both globally and within geographically defined populations. Isolation by distance was tested between the same putative populations using a Mantel Test. First, average latitude and longitude were calculated for each geographic cluster. Next, pairwise distances between the average locations were calculated using a least cost pathway analysis in the R package marmap [50]. The pathway is allowed to vary across both depth and geographic disatance. The natural log of these distances were compared to linearized pairwise genetic distance (F_ST_/ (1-F_ST_)) using the Mantel test carried out in adegenet with 1000 randomizations.

## Results

### Species Confirmation

**Fig 2:**
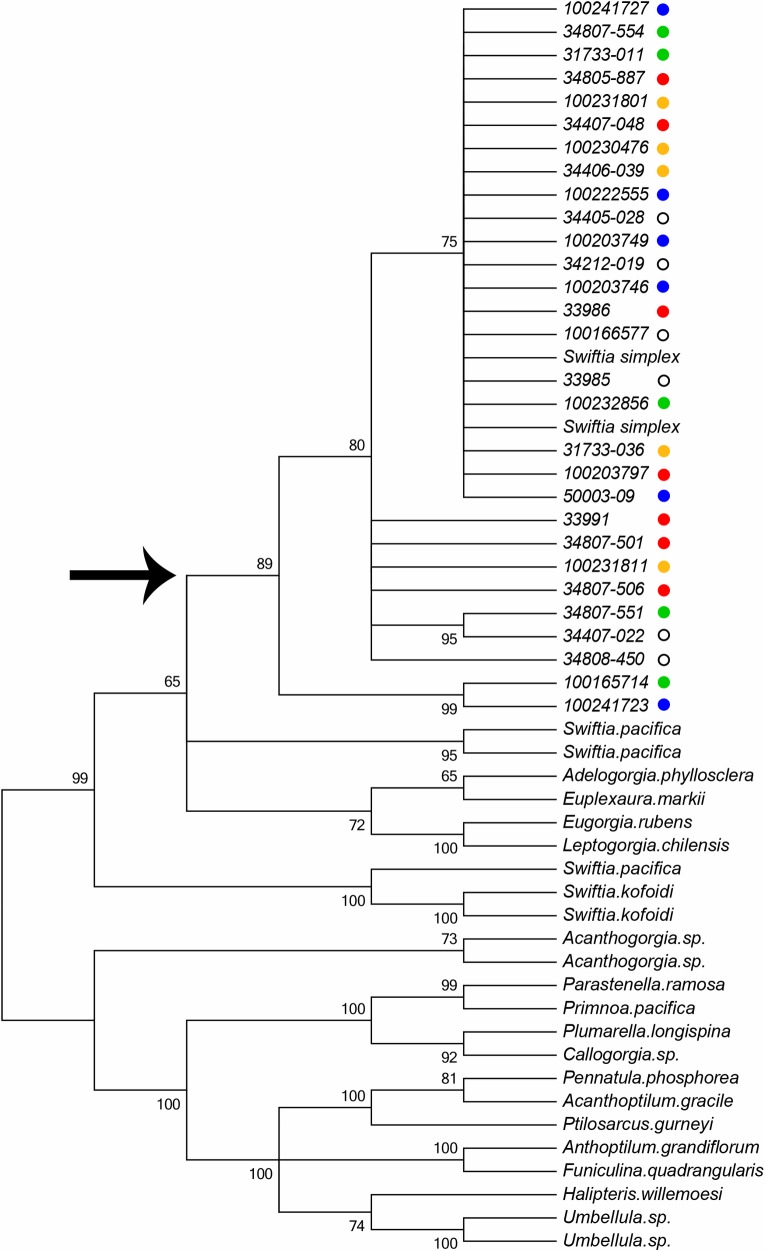
Maximum parsimony phylogeny obtained with the *MutS* sequence. The arrow indicates the branch containing all *S*. *simplex* individuals. Additional species are from our voucher collection (S1 Table). Colored dots after each individual correspond to putative population groupings indicated in Fig 1 and Table 1. Numbers at branch nodes are bootstrap values from 1,000 replicates. While there are four haplotypes of *S*. *simplex*, there is no correspondence with geographic grouping. Branch lengths are not scaled.

Mitochondrial amplicon sequence was successfully obtained for all twenty-nine individuals to check species identification. The full concatenated *MutS+igr+COI* mitochondrial sequence was obtained for thirteen of the twenty-nine individuals. Ongoing phylogenetic work carried out by our laboratory has indicated that species assignment in *S*. *simplex* is successful with *MutS* alone (data not shown), thus in the remaining sixteen individuals only the octocoral specific *MutS* gene was amplified. Successful sequence data was deposited in GenBank, see Table 1 for accession numbers. Within our samples four MutS haplotypes and a single haplotype of *igr+COI* were identified. Pairwise genetic distances among the *MutS* haplotypes were less than 1% and, following the precedent described in McFadden et al. [6], we considered this to be within the bounds of intraspecies variation. Species identification was successfully confirmed via phylogenetic analysis for the twenty-nine individuals initially included in the study (Fig 2).

### SNP Discovery and Genotyping

Twenty-six individuals yielded sufficient DNA to be included in the RAD-tag library construction. RAD-tag sequences were deposited in NCBI’s Short Read Archive (SRA) (Accession number SRR4296452). After quality filtering and demultiplexing the resulting RAD-tag sequences, three individuals had extremely low sequence counts (below 16,194 reads per individual) and were excluded from all further analysis. An average of 355,393 reads was obtained for each of the remaining twenty-three individuals, though there was considerable variation in reads per individual (ranging between 63,360 and 1,077,283 reads per-individual), likely caused by the range of DNA qualities observed on the agarose gel. Lower quality DNA can result in a loss of raw reads due to low sequence quality scores [51] (S2 Table). After running Stacks, the initial catalog was comprised of 9,707 tags containing 22,843 putative SNPs. All twenty-three individuals analyzed were successfully genotyped against the full catalog; resulting in genotypes for 17,769 SNPs after filtering for minimum coverage (see methods). After removing tags containing more than three SNPs and SNPs that genotyped in only a single individual (singletons), 4,249 SNPs remained.

At this point two subsets of data were created (see Methods) and each subset was filtered for low MAF and HWE. After these filters were applied, 4,094 SNPs remained in the n = 23 subset, and 4,186 SNPs remained in the n = 20 subset. Finally, SNPs that failed to genotype in at least 80% of individuals were removed, leaving 1,132 (n = 23) and 1,726 (n = 20) SNPs in for further analysis.

### Population Structure

Initial tests for population structure were carried out in STRUCTURE. To reduce linkage in the data, only the first locus in any tag containing more than one SNP was included, so the initial tests were run on 786 of the 1,132 (n = 23) and 1,145 of the 1,726 (n = 20) SNPs genotyped. In the data subset containing all twenty-three individuals the STRUCTURE results indicated the highest average likelihood with the lowest variance was found for K = 1 across all 23 individuals. In this situation Evanno’s test for detecting the number of clusters of individuals is not applicable [52]. To test the possible effect of missing loci, additional STRUCTURE runs examined markers genotyped in 90% (n = 406 SNPs) and 95% (n = 228 SNPs) of individuals. For the 95% data set the highest probability was again obtained for K = 1. The result of K = 1 was generally consistent in these two tests. For the 90% dataset K = 5 had a slightly higher average probability, but overall higher variance and examination of clusters generated with the K = 5 failed to find any consistent patterns. In the n = 20 data subset, the highest average likelihood with the lowest variance was again obtained for K = 1. Testing the effects of missing markers, STRUCTURE was again run on the markers genotyped in 90% and 95% of individuals. In both additional tests, average probability values were approximately equal for multiple values of K, including K = 1, but K = 1 had the lowest overall variance in both additional tests. Examination of the STRUCTURE plots for each K in both runs revealed full admixture across all individuals, indicating that K = 1 is likely the true scenario(See S1 Fig for example plots).

**Fig 3:**
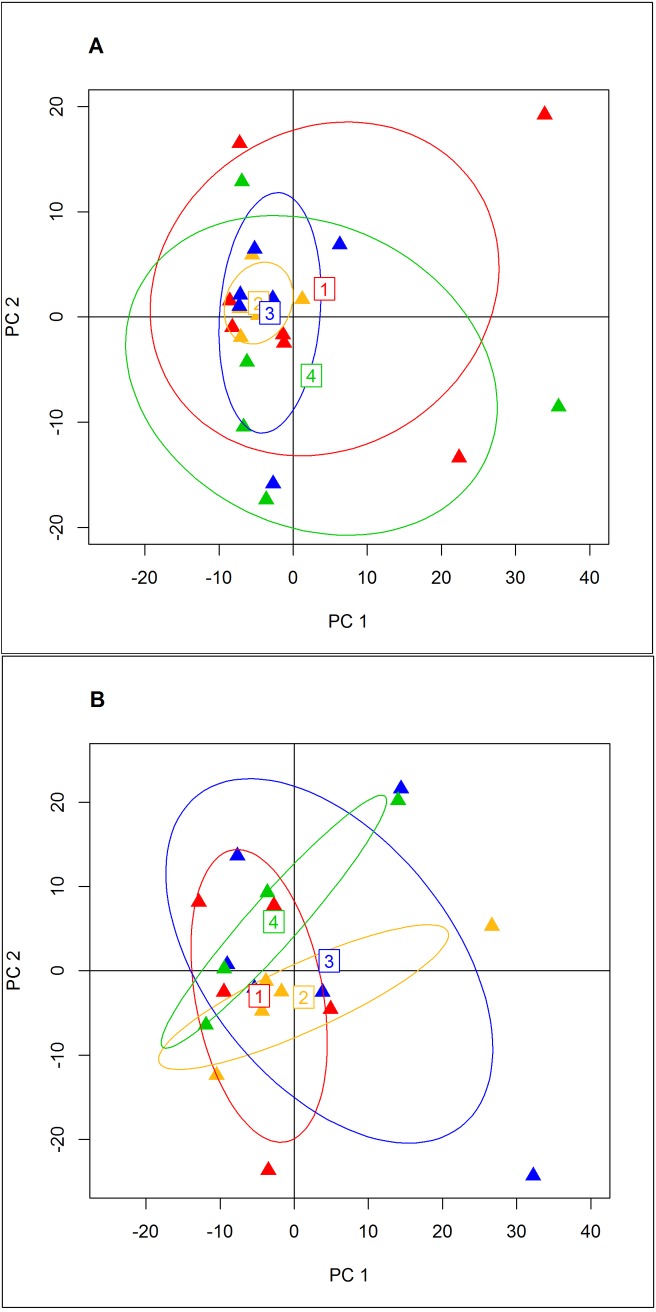
Principal components analysis on RAD-tag genotypes. Colors correspond to putative geographic population groupings in Fig 1 and Table 1. A. Includes all 23 individuals. The three outliers are the three individuals with the most missing loci. PC1 and PC2 explain 10.5% and 5.5% of the total variance respectively. B. PCA after removing the three individuals with excess missing markers. The separations along PC1 in both cases are driven by missing genotypes. After removal PC1 and PC2 explain 6.7% and 6.6% of the variance.

PCA was performed using the R program adegenet. Initial tests with all 23 individuals and the full set of 786 markers showed all four geographic groups overlapping (Fig 3A), however, three individuals showed significant separation along PC1. These three individuals (100203797, 100120887, and 100186551) have the highest percentage of missing markers (S2 Table). Running the test on the dataset with the three individuals removed, all three geographic groups were again overlapping with no discernable separation. The minor separation of four individuals along PC1 is again driven by missing markers, in this case in the individuals with the 4^th^-7^th^ highest missing marker counts in the full data set (Fig 3B). Tests with higher percentages of genotyped markers (as above) did not change the results.

As removing the three lowest individuals did not have any apparent effect on possible population structure, the population statistics were calculated on the full twenty-three individual dataset. The overall F_ST_ for the full data set was 0.0056 and was not statistically different than 0 (p = 0.30). Pairwise F_ST_ values ranged from -0.0016 to 0.0123, and none were statistically significant (p = 0.1862 to p = 0.6828) (Table 2). The results of an AMOVA across all geographic regions populations and individuals displayed much higher variance within populations than among populations. A total of 99.4% of the variation was explained by variation within populations (Table 3). Observed and expected heterozygosities varied from 0.247 to 0.259 and 0.247 to 0.271 respectively (Table 4). The results of a Mantel test for isolation by distance reported no significant relationship between genetic F_ST_ and geographic distance (p = 0.099).

**Table 2:** Pairwise F_ST_ values between populations.

	Pop 1	Pop 2	Pop 3	Pop 4
Pop 1	-	0.4057	0.4699	0.1862
Pop 2	0.0049	-	0.3783	0.3042
Pop 3	0.0032	0.0073	-	0.6828
Pop 4	0.0123	0.0078	-0.0016	-

F_ST_ values are below the diagonal, while p-values are above the diagonal.

**Table 3:** AMOVA between putative populations.

Source of Variation	d.f.	Percentage of Variation
Among population	3	0.56
Within populations	42	99.4

**Table 4:** Putative populations, sample size, observed heterozygosity (H_O_), and expected heterozygosity (H_E_) calculated over the 786 loci.

Population	N	H_O_	H_E_
Pop 1	7	0.255	0.271
Pop 2	5	0.259	0.256
Pop 3	6	0.252	0.258
Pop 4	5	0.247	0.247

## Discussion

### SNP Genotyping and Population Structure

This study represents the first genomic sequence resource and the first population level study in *Swiftia simplex*. We successfully identified and genotyped more than 1,000 SNP loci in twenty-three *S*. *simplex* individuals. Within the limited number of samples examined, we found no genetic structure amongst geographic groups spanning the entire United States West Coast region, indicating the possibility of high connectivity and possible panmixia among all regions.

The application of large numbers of SNP markers has enhanced the ability to detect previously cryptic fine-scale population structure in a number of species [31–33]. Our study successfully genotyped more than 1,000 SNPs genome-wide in *S*. *simplex*, and applied 786 of these to our population analyses. We tested the possible trade-offs between limiting the number of missing genotypes in the sample and maximizing the numbers of individuals analyzed, including between 228 (n = 23, 95% genotyped) and 1,145 (n = 20, 80% genotyped) SNPs to obtain our results. In all cases we detected no discernable population structure, indicating the likelihood of high gene flow along the U.S. West Coast; however, we cannot rule out the possibility that including samples from a larger geographic area such as *S*. *simplex* individuals from Alaska, would reveal genetic structure over a wider geographic area.

Given the difficulty of sampling deep-sea corals, the need for large numbers of individuals can be a barrier to population genetics analyses. The current study included relatively few individuals (n = 23) compared to many traditional population genetics studies; however, recent studies have shown that larger sets of markers can overcome low sample sizes in population studies[53]. In Crucian carp, *Carassius carassius*, the authors demonstrated that RAD-tag markers were able to detect finer structure among populations than microsatellites (13,189 SNPs vs. 13 microsatellites), despite including only 17.6% of the individuals genotyped with microsatellites (n = 848 with microsatellites, n = 149 with SNPs) [54]. The use of large sets of SNPs can detect structure in even smaller sample sizes. A recent simulation study demonstrated that accurate F_ST_ values can be obtained with sample sizes as low as 4–6 individuals when numbers of sufficient bi-allelic markers are included. Subsequently, the use of large numbers of SNP markers has been successfully applied to detecting population structure in biological data obtained from small samples sizes. For example, a study in seahorses successfully detected population structure among twenty-three individuals from across the United States East Coast [30]. The ability to obtain accurate population structure can be especially important for endangered or rare species. In one such study, researchers successfully used genome-wide SNPs in Aye-Aye, a highly-specialized lemur species from Madagascar, and were able to detect population structure from across the species range using just twelve individuals [30].

The cost and difficulty of obtaining deep-sea coral samples can be a barrier to large scale population studies in these organisms. In addition to using high-throughput genotyping-by-sequencing to compensate for limited sample sizes, our study successfully utilized an existing collection, obtained largely as fisheries bycatch (Table 1). The use of existing collections of deep-sea corals to obtain sufficient individuals was previously utilized with mixed success. Miller et al. [17] attempted to expand their sample sizes of nine deep sea coral species from Australia and New Zealand using a large collection of museum specimens. Unfortunately, their effort was largely unsuccessful with specific success rates dependent on taxonomic group and sample age. The authors also acknowledged the significant possibility that many of the older museum specimens may have been originally stored in formalin, or handled inconsistently, which would decrease the possibility of success. Conversely, Herrera et al. [25] successfully used specimens of *Paragorgia arborea* from various museum and laboratory collections in their study of global haplotype diversity. Our study included bycatch samples inadvertently caught during the course of regular annual trawl surveys [35], in addition to those obtained in a more targeted manner [20]. While the surveys prefer to avoid coral bycatch, some living coral is incidentally caught, and has become a significant resource for the samples in this study and for the mapping and tracking of the greater deep-sea coral community along the U.S. West Coast.

The life history traits (slow-growth, longevity) [25], and the diversity of connectivity patterns in deep-sea corals, including *S*. *simplex*, can impact their ability to recover from anthropogenic disturbances, including fisheries activities, and thus have implications for their protection and management [11, 12, 25, 27, 55]. Deep-sea corals are a critical component of the deep-sea ecosystem, creating structural habitats that support a diversity of additional species, including commercially important fisheries. As a result, in 2006, deep-sea corals were added to the Magnuson-Stevens Fishery Conservation and Management Act, to begin monitoring and protecting deep-sea coral areas [56]. Species with broad scale larval dispersal, and high degrees of connectivity are more likely to recover from disturbance than those with extremely limited dispersal patterns [57]. Thus knowing the degree of connectivity among populations is critical to management efforts such as the design of marine reserves [12, 55, 57].

Within deep-sea corals, population structure and degree of connectivity can vary over both geographic and depth scales and can be influenced by factors such as larval duration and oceanographic circulation. A handful of studies have examined a number of deep sea corals with differing dispersal capabilities, and found a range of results regarding population structure: A study examining microsatellite and mitochondrial markers in mesophotic populations of *Corralium rubrum* identified significant population structure at all spatial scales tested [55]. This strong evidence of population structure is consistent with the low larval dispersal potential in this species. Using microsatellite loci, Quattrini et al. [26] found depth dependent population structure within *Callogorgia delta* from the Gulf of Mexico. A study by Miller et al. [17] demonstrated species specific patterns of genetic diversity and connectivity in deep-sea corals from Australia and New Zealand. *Desmophyllum dianthus* had significant genetic subdivisions among populations and regions, but four additional scleractinians, including one occurring in the same family as *D*. *dianthus*, showed no evidence that groups from across Australia/New Zealand are distinct, with haplotypes shared across all included regions. All the species included in the study are believed to be seasonal gonochoric broadcast spawners, suggesting that the larval dispersal potential in *D*. *dianthus* is lower than for the other species, even within family. In *L*. *pertusa*, an isolation by distance pattern is supported across the entire geographic area (Gulf of Mexico to the Eastern North Atlantic) [16]. Examining mitochondrial haplotypes in *Paragorgia arborea*, Herrera et al. [25] found that haplotype diversity varied over the species range, and the lowest levels of haplotype diversity occurred in *P*. *arborea* individuals from the North Eastern Pacific. This diversity in the degree of genetic connectivity among corals highlights the need for additional study and the development of additional molecular tools that can be applied towards understanding the connectivity amongst deep-sea coral communities.

We have successfully developed the first large scale molecular sequence resource for *S*. *simplex*, from the United States West Coast. Our study demonstrates the apparent lack of genetic structure along the United States West Coast and suggests a high level of genetic connectivity in *S*. *simplex*. This is consistent with the expectations for a highly fecund broadcast spawner [23], and suggests that local populations of *S*. *simplex* may have the capability to recover from or recruit following disturbance, at least on a regional scale, given sufficient time for immigration of new settlers.

## Supporting Information

S1 Fig Example STRUCTURE output for the twenty-three individual dataset. STRUCTURE plots for K = 2-K = 6 generated using the full twenty-three individual dataset. No pattern was observed in for any K value, consistent with a panmictic, K = 1 population.
(TIF)

S1 Table Accession numbers for the reference individuals used in the species confirmation. (XLSX)

S2 Table RAD sequencing summary for all individuals. (XLSX)
