# Supplementary figures and images for: Large-Scale Genotyping-by-Sequencing Indicates High Levels of Gene Flow in the Deep-Sea Octocoral Swiftia simplex (Nutting 1909) on the West Coast of the United States
Source: PLoS One. 2016 Oct 31;11(10):e0165279. doi: 10.1371/journal.pone.0165279 (PMC5087884; doi:10.1371/journal.pone.0165279)

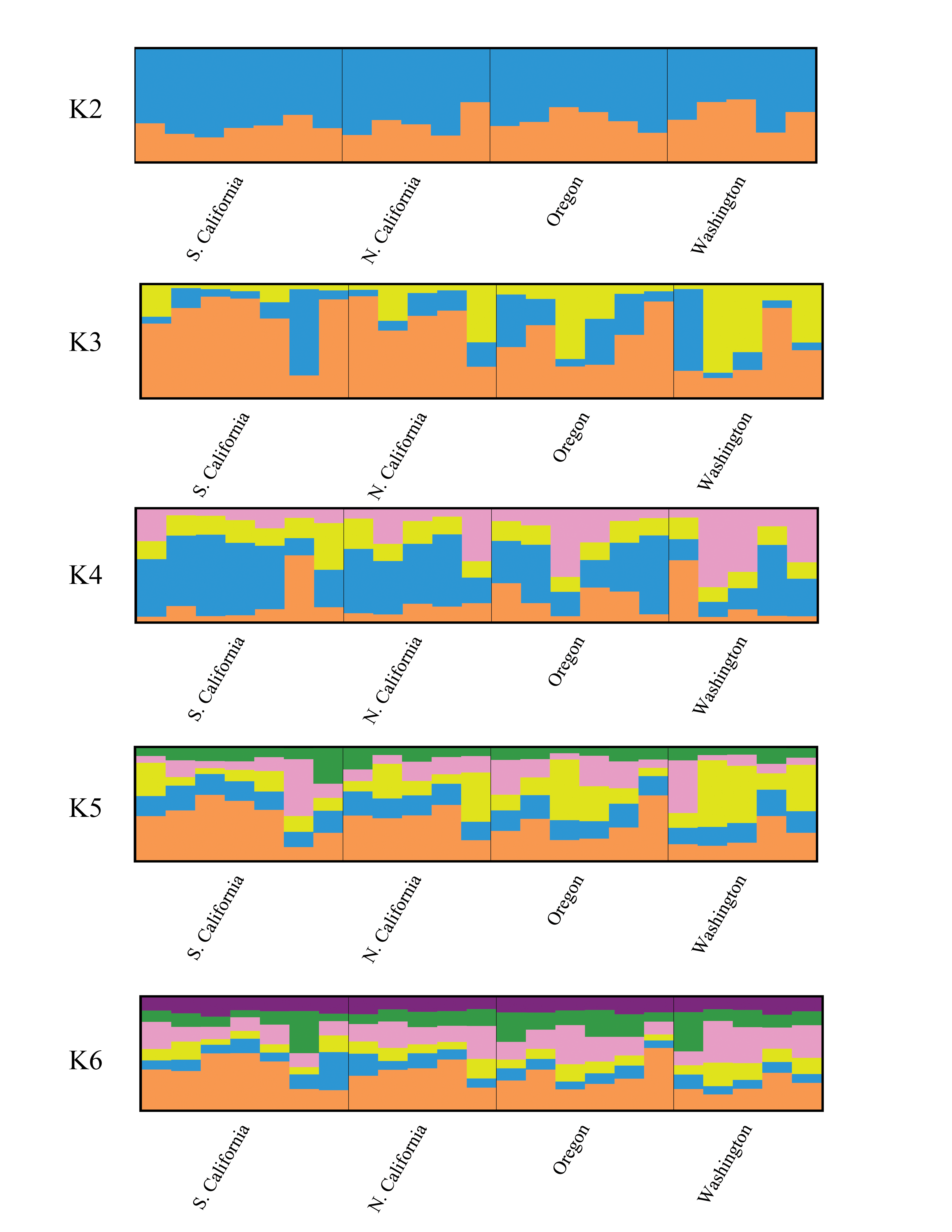

Supplement: S1 Fig — STRUCTURE plots for K = 2-K = 6 generated using the full twenty-three individual dataset. No pattern was observed in for any K value, consistent with a panmictic, K = 1 population. (TIF) [file pone.0165279.s001.tif]
